# Supplementary material for: GenoTriplo: A SNP genotype calling method for triploids
Source: PLoS Comput Biol. 2024 Sep 24;20(9):e1012483. doi: 10.1371/journal.pcbi.1012483 (PMC11452025; doi:10.1371/journal.pcbi.1012483)
Supplement: Attachment — Submitted filename: Reponses_to_reviewers_GenoTriplo.pdf [file pcbi.1012483.s001.pdf]

Dear editor and reviewers,

We thank you for your comments which help us to improve the quality of our manuscript "GenoTriplo: A SNP genotype calling method for triploids" for consideration at PLOS Computational Biology. Please find below our detailed answers.

Reviewer #1: In this paper, the authors developed GenoTriplo, a computational method to call SNP genotype for triploids. This work addressed an important research question. The proposed methods are interesting. The authors conducted comprehensive benchmark analysis to compare GenoTriplo with existing method fitPoly, and results are solid. The paper is also well written. I think this work has potential to attract general interest of the readers of PLOS Computational Biology. I just have a few minor comments and suggestions which may further improve the current manuscript. Below are my specific comments:

1. The authors compared the performance between GenoTriplo and fitPoly. When two methods provide inconsistent genotype calling results, what is the gold standard / grand truth? In practice, when the genotype truth is unknown, how can users evaluate the quality of GenoTriplo called genotypes? It would be very helpful if the authors can provide some general guidance for practitioners.

As explained in the paper, the "grand truth" is for now the human eye. That is why we visually assessed the difference of the two methods for a large number of markers. Images such as the ones in the paper were sorted based on what the observer was expecting. Users do not have a simple metric for the quality of the genotyping but they can set thresholds to ensure good genotyping. Users can also look over some markers to verify that genotyping performs well (i.e. that the clustering done by the program conforms with what we would do manually). Users can also manually change the genotype of some individuals within the package Shiny App in the visualization section. However, we don't recommend to use that feature in an extensive way as it would just result in a waste of time without strong improvement.

2. In Page 6, line 143. The coefficient 0.28 likes very ad hoc. The authors need to perform robustness analysis, and provide more details on the "trial and error assay" (line 151). When applying to different datasets, whether users need to change this coefficient 0.28? Similarly, the authors need to provide more justification on the following numbers: (1) Page 11, line 228 "0.85", (2) Page 12, line 245 "0.97", (3) Page 12, line 250 "3.4"

0.28 : added justification in Materials and Methods l. 152 to 182. This parameter is susceptible to be dependent of the ploidy level (GenoTriplo might be extended to higher ploidy levels). It has been tested so that 0.28 works for both diploids and triploids. But if there are some clustering issues, this parameter could be modulated.

0.85 : added justification l. 256 to 259

0.97 : added justification l. 275-276. CR of each marker can be found in the results; a supplementary filter can be applied by users.

3.4 : added justification l. 282-283. User can filter afterward as FLD can be found in the results.

3. The authors developed R package GenoTriplo and released it at CRAN. They also need to provide more details on the computational cost, including memory and time.

A ‘Computational cost’ section was added to the results.

4. Typo: Page 2, line 36. "One can utilize individuals" is duplicated.

Thank you for your attention. The duplication has been removed.

Reviewer #2: The SNP genotype calling method (GenoTriplo) developed for triploids is very interesting. However, the following concerns should be addressed.

Comments:

1.To better test the accuracy of genotyping calling using GenoTriplo, the authors should test the genotype calling from various populations of genetically distant and compare the results with that of single population analysis performed in the current study.

GenoTriplo was tested on triploid apple cultivars to test its robustness in response to this suggestion. See the new section ‘Validation dataset’ in the article. The luminescence data needed were extracted from Illumina SNP array to test GenoTriplo on another technology.

2.Table 2 shows that GenoTriplo identifies significantly less SNPs compared to fitPoly with increased complexity of genotypes. It indicates that GenoTriplo is less efficient in case of complex genotypes. Please provide possible explanation if possible with analysis, for using GenoTriplo over fitPloly.

As explained l. 459, 292 out of 1000 markers where fitPoly identified 4 clusters and GenoTriplo only 3 was due to a single individual genotyped as homozygous by fitPoly and as NA by GenoTriplo. This is due to the unsupervised approach of GenoTriplo which tends to assign clusters only if it is worth adding a cluster (also see M&Met). And as you can see table 3, GenoTriplo genotypes are highly concordant with fitPoly genotypes for those cases, meaning that there is a small loss of genotype accuracy. In contrast, for categories where GenoTriplo was best, fitPoly had low concordance with GenoTriplo meaning a higher loss of genotype accuracy. That is clearly in favour of GenoTriplo use rather than fitPoly. See l. 459 to 464.

3.Please correct repetitive sentences from the author summary section.

Done
