# Supplementary material for: GenoTriplo: A SNP genotype calling method for triploids
Source: PLoS Comput Biol. 2024 Sep 24;20(9):e1012483. doi: 10.1371/journal.pcbi.1012483 (PMC11452025; doi:10.1371/journal.pcbi.1012483)
Supplement: Attachment — Submitted filename: Reponse_reviewer_last.docx [file pcbi.1012483.s002.docx]

Dear editor and reviewers,

It seems that the only additional concern that was not fully address in our second draft concerned the data availability as the reviewer 2 answered « NO » to the question « Have the authors made all data and (if applicable) computational code underlying the findings in their manuscript fully available ? ».

Indeed the validation dataset was missing for apple luminescence signals as we were not the owners of this dataset. We got the authorization to add the apple dataset under the following doi <https://doi.org/10.57745/7IMQDS>.

In the third draft, the only changes that have thus been done is to add this information and to acknowledge all people and associations that allowed us to give access to the apple genotypes.

Best regards,

Florence Phocas
